# Behavioral evidence for a magnetic sense in the oriental armyworm, *Mythimna separata*

**DOI:** 10.1242/bio.022954

**Published:** 2017-01-26

**Authors:** Jingjing Xu, Wei Pan, Yingchao Zhang, Yue Li, Guijun Wan, Fajun Chen, Gregory A. Sword, Weidong Pan

**Affiliations:** 1 Beijing Key Laboratory of Bioelectromagnetics, Institute of Electrical Engineering, Chinese Academy of Sciences, Beijing 100190, China; 2 Department of Electrical Engineering, University of Chinese Academy of Sciences, Beijing 100049, China; 3 Department of Entomology, College of Plant Protection, Nanjing Agricultural University, Nanjing 210095, China; 4 Department of Entomology, Texas A&M University, College Station, TX 77843, USA

**Keywords:** *Mythimna separata*, Flight simulation, Orientation, Magnetic sense, Magnetic compass

## Abstract

Progress has been made in understanding the mechanisms underlying directional navigation in migratory insects, yet the magnetic compass involved has not been fully elucidated. Here we developed a flight simulation system to study the flight directionality of the migratory armyworm *Mythimna separata* in response to magnetic fields. Armyworm moths were exposed to either a 500 nT extreme weak magnetic field, 1.8 T strong magnetic field, or a deflecting magnetic field and subjected to tethered flight trials indoors in the dark. The moths were disoriented in the extreme weak magnetic field, with flight vectors that were more dispersed (variance=0.60) than in the geomagnetic field (variance=0.32). After exposure to a 1.8 T strong magnetic field, the mean flight vectors were shifted by about 105° in comparison with those in the geomagnetic field. In the deflecting magnetic field, the flight directions varied with the direction of the magnetic field, and also pointed to the same direction of the magnetic field. In the south-north magnetic field and the east-west field, the flight angles were determined to be 98.9° and 166.3°, respectively, and formed the included angles of 12.66° or 6.19° to the corresponding magnetic direction. The armyworm moths responded to the change of the intensity and direction of magnetic fields. Such results provide initial indications of the moth reliance on a magnetic compass. The findings support the hypothesis of a magnetic sense used for flight orientation in the armyworm *Mythimna separata*.

## Abstract (precis)

**Summary:** Responses to changes in the intensity and direction of magnetic fields in the armyworm moth (*Mythimna separata*) indicate a reliance on a magnetic compass for flight orientation.

## INTRODUCTION

The geomagnetic field is an environmental cue that varies predictably across the surface of the globe. It provides animal with two potential types of information. The positional information of the geomagnetic field can be used as a magnetic map, whereas the directional information can be used as a magnetic compass (Lohmann et al., 2007). The magnetic intensity and inclination can serve as a component of the navigational ‘map’, and specific magnetic conditions of local regions may act as ‘sign posts’ (Wiltschko and Wiltschko, 2005). The magnetic compass included the polarity compass and the inclination compass (Johnsen and Lohmann, 2005). Organisms have evolved sensory systems to detect and exploit these cues in their environment to use information about the geomagnetic field to guide their movements in ways that enhance fitness (Lohmann et al., 2007).

Many animals use the directional information from the Earth's magnetic field for orientation and navigation (Muheim et al., 2016). Magnetoreception, the ability of an organism to detect a magnetic field, is phylogenetically widespread. Diverse organisms ranging from bacteria to vertebrates showed behavioral responses to variation in magnetic fields (Wiltschko and Wiltschko, 2005). Magnetic compass orientation has been known in birds for more than 40 years, such as European robins (Wiltschko and Wiltschko, 1972), homing pigeons (Walcott and Green, 1974) and sanderlings (Gudmundsson and Sandberg, 2000). It is reported that anthropogenic electromagnetic noise could disrupt magnetic compass orientation in a migratory bird (Engels et al., 2014). Loggerhead sea turtle hatchlings have been demonstrated to distinguish between different magnetic inclination angles and field intensities and possess the minimal sensory abilities necessary to approximate global position (Lohmann and Lohmann, 1994,^1996^). There is also the evidence for a robust magnetic compass response in C57BL/6J mice (Muheim et al., 2006). There are several examples among invertebrates as well. The nematode *Caenorhabditis elegans* exhibited changes in vertical burrowing movements as the magnetic pole was reversed (Vidal-Gadea et al., 2015). The use of a magnetic compass was shown to be involved in the migration of the monarch butterfly *Danaus plexippus* (Guerra et al., 2014) and the migratory butterfly *Aphrissa statira* (Srygley et al., 2006). Spontaneous magnetic orientation in larval *Drosophila* shared properties with learned magnetic compass responses in adult flies and mice (Painter et al., 2013). The orientation of a caged nocturnal moth *Noctua pronuba* was reversed in a magnetic field whose net effect was nearly a mirror image of the geomagnetic intensity and direction (Baker and Mather, 1982). The termites, *Amitermes meridionalis* aligned mound cells along the existing axis of the mound and the cardinal axes of the horizontal component of the applied magnetic field (Jacklyn and Munro, 2002). It has been reported that ants *Formica rufa* L. exhibited a magnetic compass response (Camlitepe and Stradling, 1995)

In the present study, we tested whether the nocturnal flight orientation of the armyworm *Mythimna separata* was affected by variation in magnetic fields. The armyworm is a kind of migratory pest in Asia and Australia (Sharma and Davies, 1983) that periodically causes serious damage on sorghum, pearl millet, rice, maize, wheat and sugarcane (Sharma et al., 2002). Heavy crop losses because of the armyworm have been reported in India, Bangladesh, China, Japan, Australia and New Zealand (Sharma and Youm, 1999). Its long-distance migratory behavior has been well documented in Asia (Koyama, 1970; Oku and Koyama, 1976). During the autumn migration, armyworm moths flew at the altitudes of 50-500 m, with a displacement speed of 4-12 m/s (Chen et al., 1989). The armyworm moths have been observed to exhibit directional movement while flying in large numbers in the night sky with extremely low visibility. Whatever the wind direction was, armyworm moths flew toward the southwest during autumn, and toward the north or northeast during spring (Chen et al., 1989). Other than the wind, it seemed that some cues, probably a geomagnetic or celestial compass guided the heading of these moths during long-distance migration as in diurnal migratory butterflies (Perez et al., 1997; Oliveira et al., 1998; Srygley et al., 2006). The behavior of directionality has evolutionary meaning with directing insects toward favorable ecological regions for reproduction or surviving winter. Common orientation among individuals in flying high-density groups may lead to landfall in a relatively small area (Wolf et al., 1995), resulting in rapid local insect outbreaks due to mass immigration. Although the phenomenon of directional migration has been studied intensively, the intrinsic mechanisms that underpin navigation behavior remain largely unknown. To identify the potential magnetic sensory mechanism involved in the migratory orientation of armyworm moths, we developed a flight simulator and tracking system (Shen et al., 2012; Wang et al., 2013). In the complete dark, the tethered flights of armyworm moths were investigated indoors in geomagnetic field (GMF), 500 nT extreme weak magnetic field (WMF), a 1.8 T strong magnetic field (SMF), east-west magnetic field (EWMF), and south-north magnetic field (SNMF) to determine whether the intensity and direction of the magnetic field could affect their flight orientation behavior.

## RESULTS

### Flight orientation distribution of a single armyworm moth

**Fig. 1.**
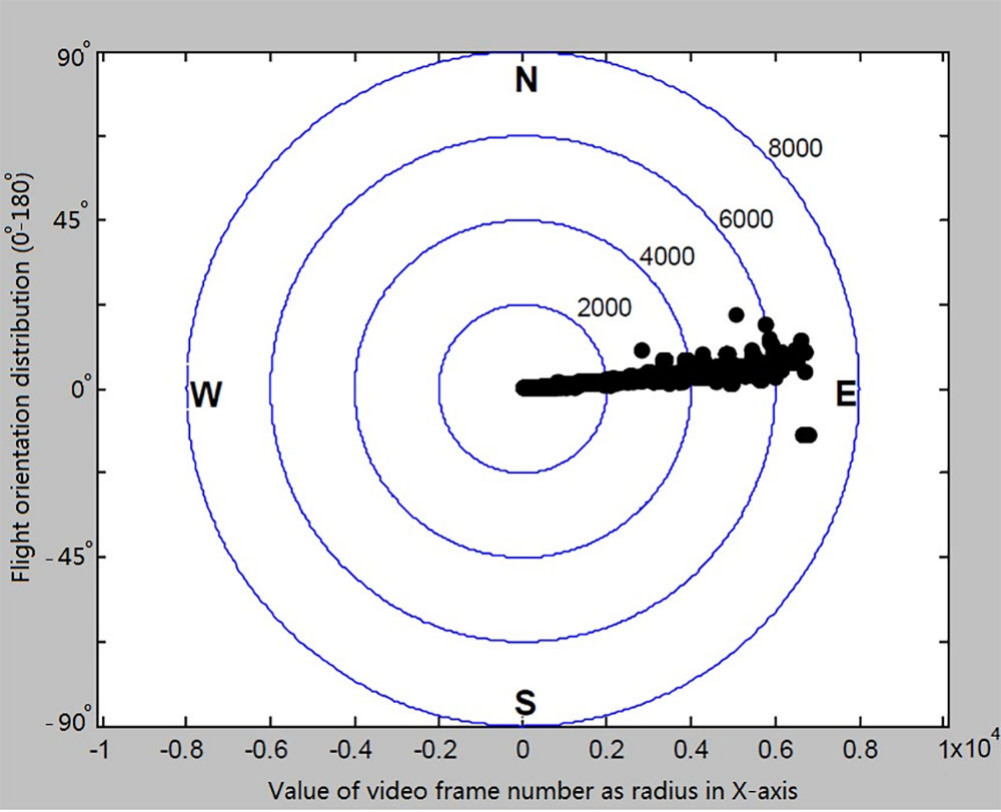
**Flight orientation analysis for an *armyworm moth* in the geomagnetic field in the spring.** The total frame number *N*=6878. The center coordinates (*x_0_*, *y_0_*) of directional indicator disk=(290, 242) and the coordinates of *N* position (*x_N_*, *y_N_*)=(356, 96). Directions are mean±1 circular standard deviation. Orientation distribution plotted using the value of video frame number as the radius. The average heading direction was calculated as 10.4±4.19°. East=0° and west=180°.

To detect the availability and reliability of the self-made system, we first tested the tethered flight of a moth in the spring. Each single moth was videoed using the horizontal camera and vertical infrared cameras for 30 min in the darkness and the first active 10 min of flight was used for data processing. The heading direction was recorded from each video frame and analyzed by using the records of whole video frames. Provided the starting position of the moth as (0, 0) and running time *t* with the speed of *v* and direction angle of *α1*, the acquired position was resumed as a new origin to continue running next *t* time with the speed of *v* and heading direction of *α2*. The procedure was repeated until the entire tracking time *T* was achieved. The orientation distribution of individual adult moth was achieved using the angle of each coordinate point relative to the *x* axis with the number of video frames as the radius. An example of the virtual flight trajectory reconstructed for an individual adult moth is as shown in Fig. 1. The orientation distribution of individual adult moth was achieved as shown in Fig. 1. The mean resultant vector direction was calculated as 10.4±41.97°.

### Flight behavior in the geomagnetic fields and extreme weak magnetic field

**Fig. 2.**
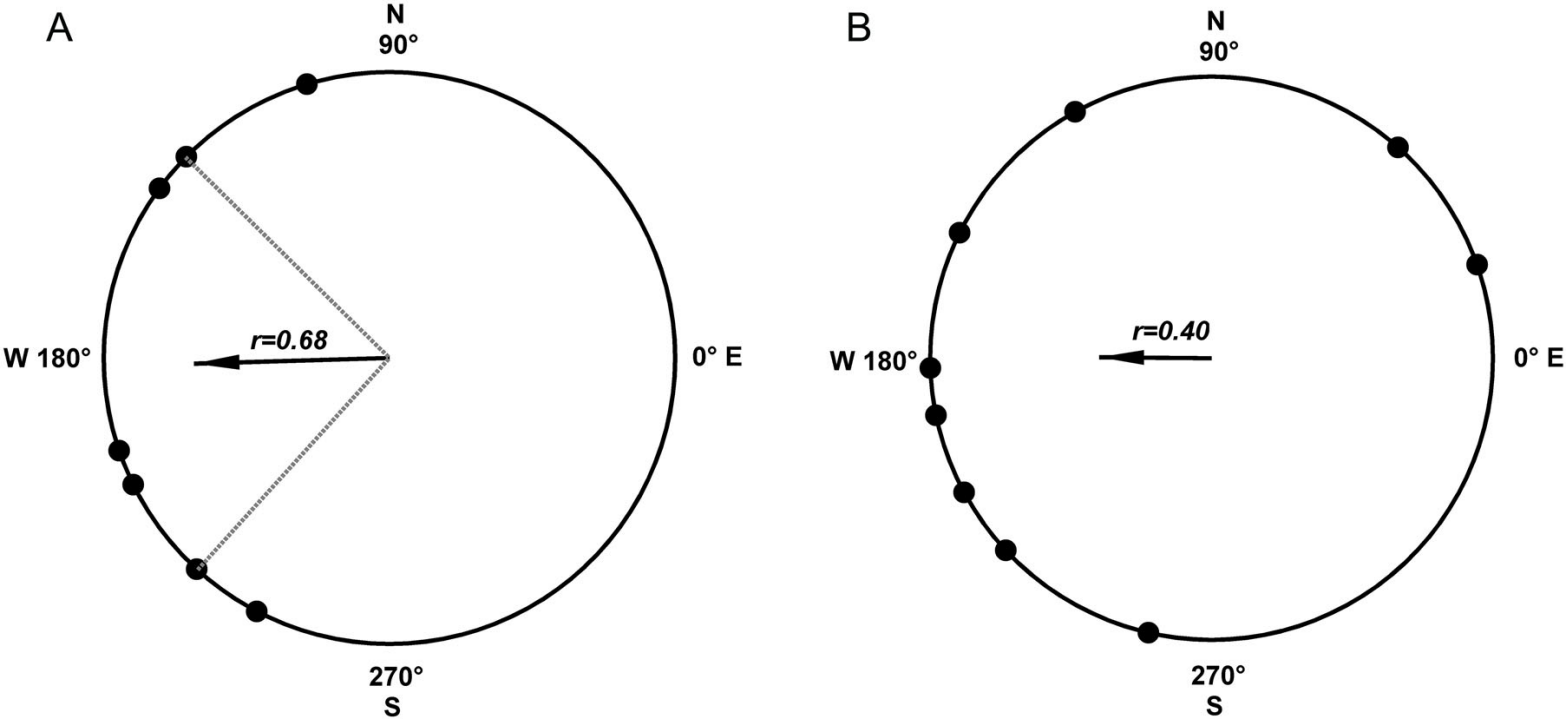
**Orientation distribution of armyworms.** Orientation distribution of armyworms in (A) the geomagnetic field and (B) extreme weak magnetic field. The black dots on the circles represent the heading direction of one armyworm moth. The black arrows represent the mean vector bearings, with the length of the arrow proportional to the resultant vector length (*r*). Dashed grey lines show the 95% confidence intervals for mean vectors that are significant by the Rayleigh test (*P*<0.05). The armyworm moths exposed to the GMF commonly oriented with the mean direction of 181.6°, and the lower/upper 95% confidence limit was 135.5°/227.8° (Rayleigh test, *P*<0.05). The armyworm moths exposed to WMF exhibited dispersed flight directions without 95% confidence intervals.

There is no obvious difference of experimental feature sound, vibration or coil system identified between GMF and WMF groups. GMF (*n*=7) and WMF (*n*=9) resulted in mean resultant vectors pointing towards 181.6° and 179.5°, respectively. No statistical significance was observed between the mean directions of two groups. As shown in the angular distribution diagram (Fig. 2), the flight angles distribution was concentrated in GMF but dispersed in WMF. The circular statistical variances of the flight orientation of armyworm moths in the GMF and WMF were 0.32 and 0.60, respectively. In addition to the larger variance, the flight angles of the armyworm moths in the WMF showed no 95% confidence intervals, which also implied dispersion. The resultant vector lengths of flight angles for the GMF group and the WMF group were 0.68 and 0.40, respectively. The closer the resultant vector length is to the value 1.0, the more concentrated the flight angles are around the mean direction (Berens, 2009). The flight angles of the GMF group displayed a common mean direction (Rayleigh test, *P*<0.05), while the flight angles of the WMF group were distributed uniformly around the circle (Rayleigh test, *P*>0.05). The skewness values showed that the flight angular symmetry of the WMF group was poorer than that of the GMF (Table 1).

**Table 1.**
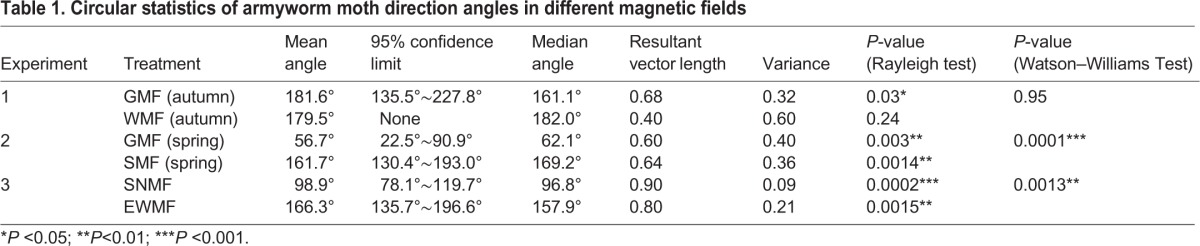
**Circular statistics of armyworm moth direction angles in different magnetic fields**

### The deflection of flight direction after exposure to the 1.8T strong magnetic field

**Fig. 3.**
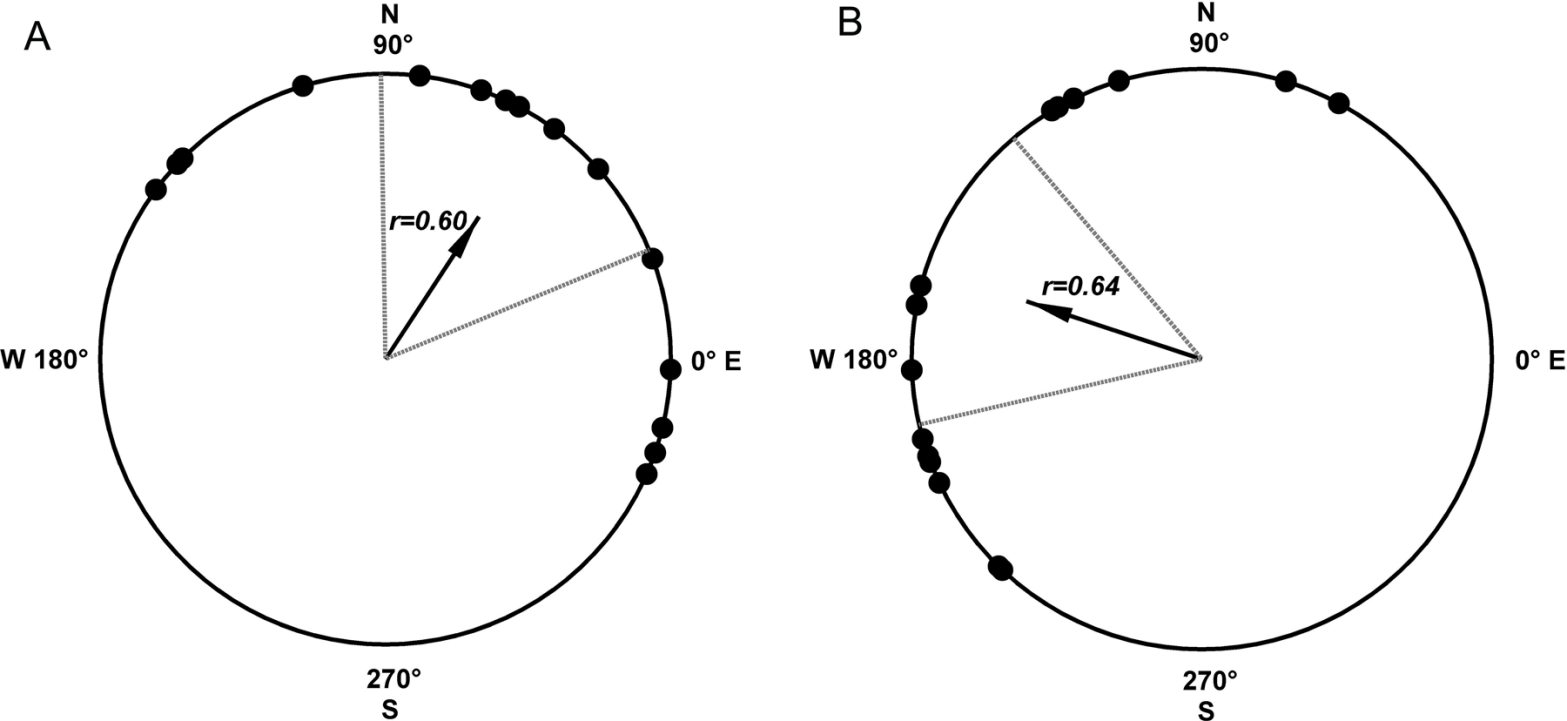
**The flight orientation deflection after magnetized by 1.8 T static magnetic field.** (A) Armyworm moths in the geomagnetic field commonly oriented with the mean direction of 56.7°, and the lower / upper 95% confidence limit was 22.5°/90.9° (Rayleigh test, *P*<0.05). (B) Armyworm moths magnetized by 1.8 T high stable magnetic field commonly oriented with the mean direction of 161.7° with the 95% confidence intervals of 130.4°-193.0°.

A total of sixty healthy adult moths were tested, of which 30 moths could fly continuously and effectively (sex ratio 1:1). The thirty moths were randomly divided into two groups (SMF versus GMF) for the experiments. The flight directions of these fifteen moths (each group) are shown in Fig. 3. Both of the two groups (SMF versus GMF) showed the common orientation with respective significant directionality (Rayleigh test, *P*<0.05). The mean angle of the SMF group was 161.7° with the 95% confidence intervals of 130.4°-193.0° while the mean angle of the GMF group was 56.7° with the 95% confidence intervals of 22.5°-90.9°.The mean directional angles of the SMF group showed a significant 105° clockwise deflection in comparison with those of the GMF group (Watson–Williams test, *P*<0.01). As shown in Fig. 3, the flight directions of armyworm moths in the SMF group distributed in the 2nd, 3nd and 4th quadrants, whereas those in the GMF group distributed in the 1st, 2nd and 3nd quadrants. The variances were 0.40 in GMF and 0.36 in SMF, and the resultant vector lengths were 0.60 in GMF and 0.64 in SMF (Table 1). The approximate variance and resultant vector indicated that the pretreatment by the SMF had no effect on the dispersion of the flight angular distribution, but affected the flight direction. It is important to note that the WMF and SMF experiments were performed in different seasonal time points at which the flight orientation of the moths were different, hence the difference in flight directions between the two GMF groups in the two experiments (Table 1).

### The flight orientation in the east-west magnetic field and the north-south magnetic field

**Fig. 4.**
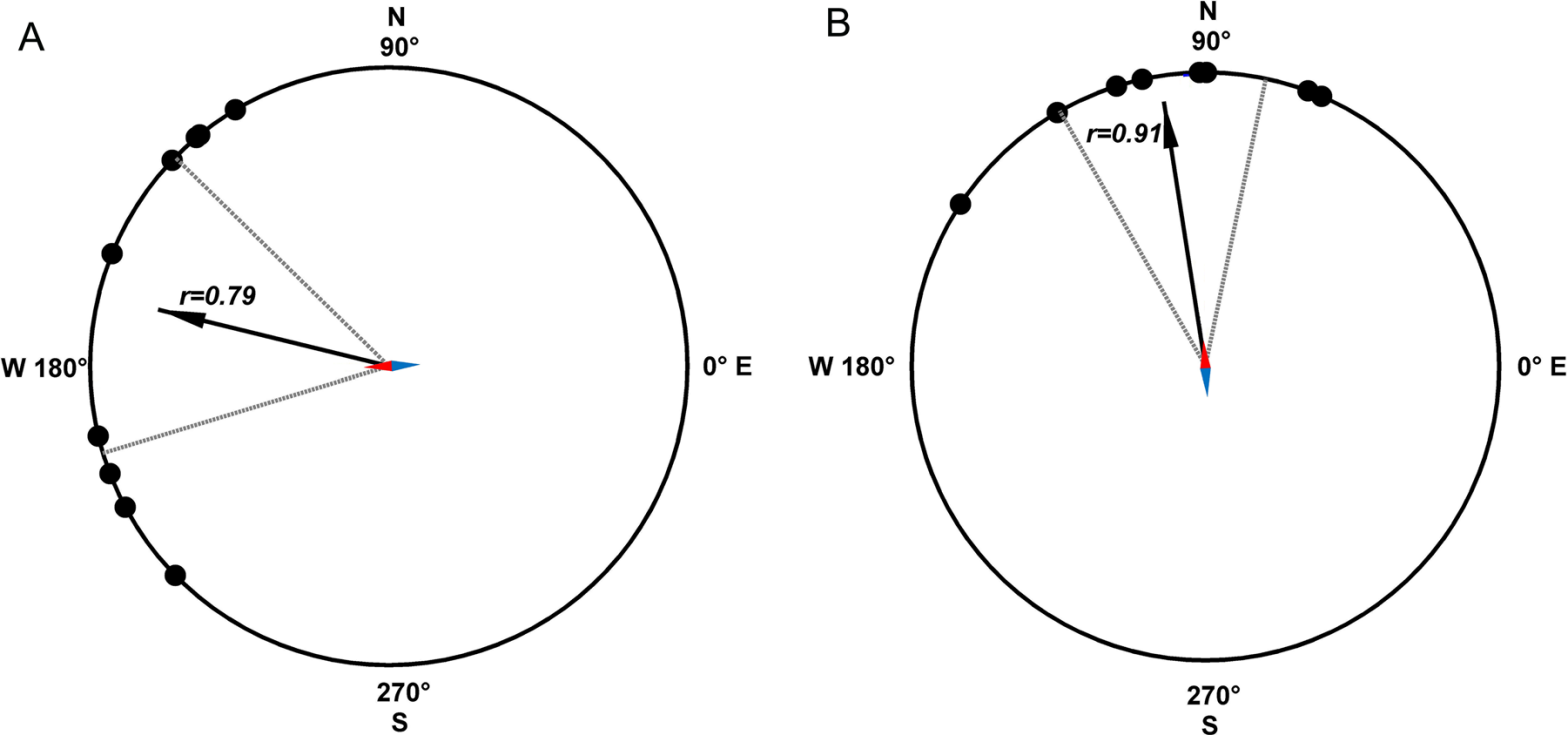
**Flight orientation varied with the direction of magnetic field.** (A) Armyworm moths in the north-south magnetic field exhibited common orientation with the mean direction of 98.9°, and the lower/upper 95% confidence limit was 78.1°/119.7° (Rayleigh test, *P*<0.05). (B) Armyworm moths in the east-west magnetic field exhibited common orientation with the mean direction of 116.3°, and the lower/upper 95% confidence limit was 135.7°/196.9° (Rayleigh test, *P*<0.05). The magnetic needle at the center of the circle shows the direction of the magnetic field.

We investigated whether the armyworm moths could sense magnetic field lines by testing the orientation of individual moths in magnetic fields with different directions. When either of the horizontal magnetic field lines was artificially shielded by the Helmholtz coil system, the moths changed their flight direction preference accordingly. The geomagnetic field intensities at the test location were measured as 16.24 μT in the north-south direction and 6.42 μT in the east-west direction. The mean direction, as shown in Fig. 4, changed with the direction of magnetic axis and distributed on the right side of the magnetic axis. The moths of both groups flew with significant directionality (Rayleigh test, *P*<0.01), which was also demonstrated by the resultant vector length closed to 1 and the variance closed to 0. The mean direction of the SNMF group was 98.90°, forming a 12.66° angle with the north-south magnetic axis direction. The mean direction of the EWMF group was 166.25°, forming a 6.19° angle with the east-west magnetic axis direction. The resultant vector lengths were 0.91 in SNMF and 0.79 in EWMF, and the variances were 0.09 in SNMF and 0.20 in EWMF (Table 1). The flight angular distributions in different magnetic fields differed significantly from each other (Watson–Williams test, *P*<0.01).

## DISCUSSION

Previous studies investigating the effect of magnetic fields on orientation considered very short distance walking, jumping and flying movements in an arena by migratory birds and butterflies (Beason and Nichols, 1984; Perez et al., 1999), which differed specifically from our study in terms of migratory flight behavior. In general, two basic methods that are currently applied to study the flight orientation of insects are to track free-flying insects outdoors or observe the orientation of tethered flight indoors (Riley et al., 1996; Guerra, 2014). Radar detection was suitable for field observations on a large scale outdoors, but do not involve responses to experimental manipulations using artificial magnetic fields (Drake, 2002). Mark-release-recapture methods have been used to collect starting and end point data, but do not provide information about movement pathways (Kuussaari et al., 1996). A flight simulator has been used to study the magnetic compass in monarch butterfly migration (Mouritsen and Frost, 2002; Guerra et al., 2014). In this study, we employed the flight simulation system to study orientation the armyworm moth in artificial magnetic field environments.

It was reported that the magnetic fields could affect the growth and development of organisms. For example, magnetic shielding induces early developmental abnormalities in the newt, *Cynops pyrrhogaster* (Asashima et al., 1991). Egg and nymph development of the brown planthopper, *Nilaparvata lugens,* was delayed by exposure to the near-zero magnetic field (Wan et al., 2014). Similar results have also been observed in plants as the pretreatment of seeds by the magnetic field enhanced germination, growth, and photosynthesis in soybean (Shine et al., 2011). Besides the growth and development, the effect of the magnetic field on the animal orientation behavior has also been studied previously. Several researchers have reported the effects of strong magnetic fields with different intensities on the flight behavior of insects. The intensity of the magnetic field varied in a big range. Redstarts, *Phoenicurus phoenicurus*, can orient in a true-zero magnetic field of ±50 nT (Mouritsen, 1998). The magnetic field strength of 10 μT may be close to the threshold of magnetoreception for Chinese noctule, *Nyctalus plancyi* (Tian et al., 2015). After exposed to a magnetic field of 0.4 T, monarch butterflies were completely disoriented (Perez et al., 1999). Neotropical migrating butterflies experimentally exposed to a strong magnetic field of 0.75 T were significantly more dispersed than those of control butterflies (Srygley et al., 2006). The threshold of magnetoreception varied for different species.

In our study, armyworm moths exhibited common orientation in the geomagnetic field, while no significant common orientation was observed among moths in the extreme weak magnetic field of 500 nT. These results supported the hypothesis that the armyworm moth flight behavior was influenced by the Earth's magnetic field. In other word, the existence of the earth's magnetic field was necessary for the flight orientation in armyworm. Our results hinted the threshold of magnetoreception for armyworm moths maybe bigger than 500 nT. The moths still exhibited common orientation in the magnetic field of 1.8 T, but their flight direction was clockwise deflected by 105°. In the previous reports, the common orientation of the armyworm moths disappeared in a strong magnetic field, which was three times the normal geomagnetic field (about 1.5×10^−4^ T in intensity) (Gao et al., 2014). In our study, however, the strong magnetic field used was 1.8 T in intensity, which was approximately 12,000 times the intensity in Gao's reports (2014). Under these conditions, the moths still exhibited common orientation similarly as described in Gao's reports (2014), but their flight direction was totally deflected by about 90°. As the magnetic fields were suggested to impose effects on organisms in a nonlinear way within a narrow functional range (Wiltschko and Wiltschko, 2005), the deflected flight orientation of the armyworm moths in the 1.8 T strong magnetic field may be attributed to magnetic nonlinear intensity-dependent effects. Here, we speculated such a strong magnetic field of 1.8 T may magnetize the moths and trigger the deflection of orientation. There may be a threshold of magnetic field intensity in the range of 0.75-1.8 T. The strong magnetic field of below the hypothetical threshold could disorient the flight of the moths, while the strong magnetic field of above the hypothetical threshold could deflect the common orientation. Indeed, this hypothesis requires further detailed experiments to verify.

The inclination compass worked when the vertical component of the geomagnetic field was reversed. For example, the mealworm beetle *Tenebrio molitor* significantly turned their preferred direction by 180° when the vertical component was reversed (Vácha et al., 2008). It has been reported that birds have a magnetic inclination compass (Wiltschko et al., 1993). Birds could not distinguish between north and south by the polarity of the geomagnetic field, but could distinguish poleward and equatorward movement by the inclination of the field lines (Wiltschko and Wiltschko, 1996). Migrant monarch butterflies also possessed a magnetic inclination compass to help guide their flight equatorward in the fall (Guerra et al., 2014). Here our results suggested the moths changed their heading direction according the deflection of the magnetic field. The moths consistently oriented in the direction of the magnetic field. The armyworm migrates in the dark; it appears unlikely that a light-based mechanism of magnetoreception is at work here. Although the navigational mechanism and genetic control of migratory flight directions are not well understood, a magnetic compass appears to be a plausible explanation.

Taken as a whole, our study revealed the sensitivity of *Mythimna separata* armyworm moths to magnetic fields. We found for the first time that the armyworm moths disoriented in the extreme weak magnetic field of 500 nT, and shifted their heading direction after exposure by 1.8 T strong magnetic field and according the deflection of the magnetic field. The moths showed behavioral responses to variations in magnetic field intensity and magnetic field direction. Many animals from diverse lineages can detect magnetic fields, but little is known about how they do so. Knowledge of the magnetic response behavior in the armyworm moths opens a new system for evaluating both the molecular and genetic mechanisms of magnetoreception that may ultimately be applied to managing this key migratory pest.

## MATERIALS AND METHODS

### Insect stocks

The insects were reared in an incubator at 24±1°C under a photoperiod of 14:10 h (L:D) with 75±5% humidity. The eggs of armyworm moths were provided by Qin in the Institute of Zoology, Chinese Academy of Science (116°39′ N, 40°00′ E). The eggs were kept in a box covered with a piece of wet gauze. After the eggs hatched, the larvae were raised from the 1st to 5th instar in open plastic boxes and fed with fresh maize leaves. As the insects grew into the sixth instar, they were transferred to transparent jars (11 cm in diameter and 12 cm in height) sealed with pieces of gauze for pupation and eclosion. After ecolsion, the adult moths were fed with 10% honey water. The 3-day-old moths of male moths and female moths (unmated) were randomly selected for experiments.

### Magnetic field devices

**Fig. 5.**
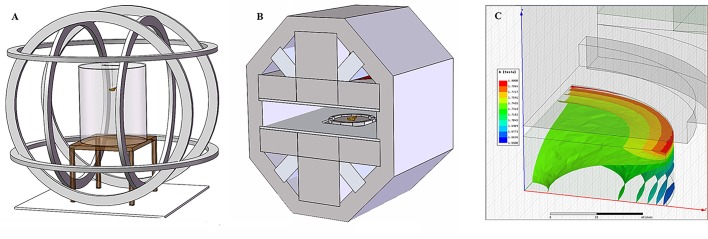
**Magnetic field generating device.** (A) Helmholtz coil system. The coil system consists of three independent coil pairs arranged orthogonally, with each coil powered by its own power supply. The near-zero magnetic field (an average intensity of 500 nT) was generated in the center spherical space (diameter=30 cm). East-west magnetic field and north-south magnetic field could also be generated in the device by controlling the power supply in either of the vertical coil pairs. (B) 1.8 T permanent magnet. It generates a static magnetic field of about 1.8 T in the center space. (C) Magnetic field distribution showing one-eighth of the central cylinder space in the permanent magnet. The magnetic field is in the *y*-axis direction (upward in the figure). Distributions for *y*-*z* plane, *z*-*x* plane and *x*-*y* plane are symmetrical.

The Helmholtz coil systems (Fig. 5A) were manufactured as described by Kovacs et al. (1997) to generate the expected magnetic fields. In this study, WMF and SNMF/EWMF were generated by three pairs of Helmholtz coils (Fig. 5A). Each coil was made up of two sub-coils that produced the same magnetic intensity. For WMF, an average intensity of ∼500 nT was produced at a center spherical space (300 mm×300 mm×300 mm). For the deflecting magnetic field, a pair of coils was used to offset the horizontal component of the magnetic field (i.e. east-west magnetic field component and north-south magnetic field component) to produce the net north-south and east-west magnetic fields, respectively. In the GMF control group, the currents in the two sub-coils flowed in opposite direction and the coils could not produce a magnetic field, but still produced the same amount of heat with the experiment groups. The magnetic flux density was measured using a fluxgate magnetometer (CTM-5W01B, National Institute of Metrology, China, sensitivity: ±1 nT) before and after the experiment. Magnetic field parameters at the position of the tethered armyworm moths during flight simulator trials (horizontal and vertical field components) were also measured using a fluxgate magnetometer.

A permanent magnet system was designed and constructed to generate a SMF of ∼1.8 T field intensity, which was approximately 3.6×10^4^ times the geomagnetic field (Fig.5B,C). The hard magnetic material used in the system is NdFeB and the material for pole pieces is electric pure iron with total system weight of less than 800 kg. At the outer yokes and triangle yoke blocks, ordinary low-carbon steel was used as soft magnetic material. For consistency, all tested armyworm moths were exposed to the magnetic field individually with the same body direction for 20 s. The magnetic induction lines were perpendicular to the body axis with the North Pole on the left. Then, the moths were kept unrestrained in the container outside the magnetic field for 5 min for quiescence and placed carefully inside the field to maintain orientation during exposure.

### Flight simulator and tracking system setup

**Fig. 6.**
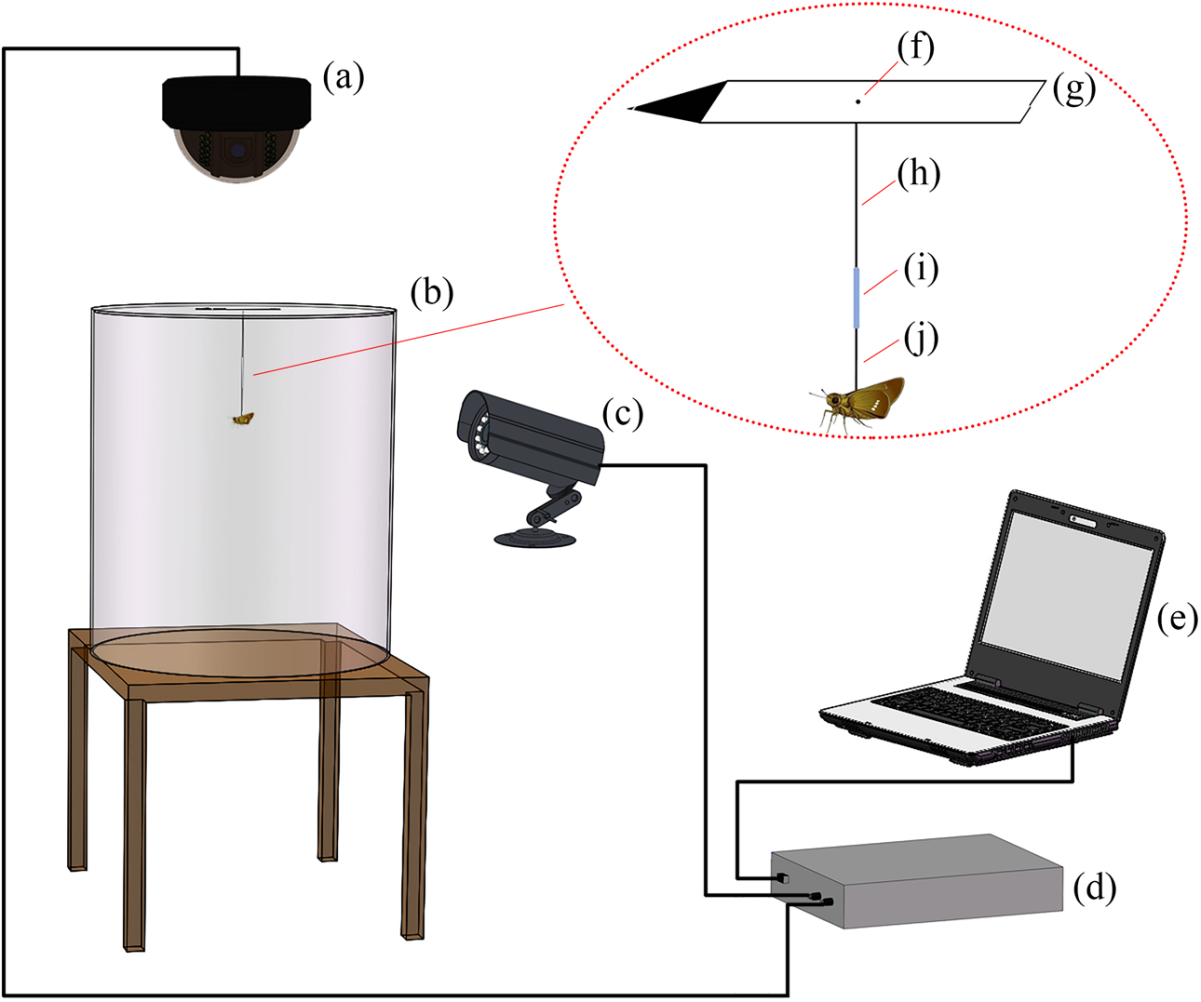
**Schematic diagram of the tethered flight system.** (A) The vertical camera; (B): the flight simulator arena; (C): the horizontal camera; (D): the video acquisition card; (E): the computer. The plastic cylinder arena (B) was 35 cm×40 cm. The handmade flight simulator consisted of five parts. The fixed wheel (F) was three glued nuts (diameter=1 mm) with the lead lazy arm (H) inserted underneath. A piece of white cardboard was cut into an arrow shape as the directional pointer, and the arrow head point was painted black as a tracking mark (G). The coupler (I) was made of the wire sheath to link the lead lazy arm (H) and the aluminium rod (J).

The flight simulator was placed in the Helmholtz coil system to tracking the flight direction of moths. The tracking platform, as illustrated in Fig. 6, consisted of a vertical infrared camera, a horizontal infrared camera, an acrylic cylinder arena (35 cm diameter and 40 cm height), a custom-made flight simulator system, a video data acquisition card and a computer. The custom-made flight simulator was comprised of a directional pointer, a lazy arm made of lead core (0.7 mm diameter), an installation rod made of aluminum wire which was used to connect the moth to the flight simulator, and a detachable conductor coupler which was used to connect the installation rod to the lazy arm. The end of installation rod was bent to expand the contact surface area with insects' dorsum. The fixed wheel, made of three screw nuts stuck together by cyanoacrylate adhesive, was fixed through an aperture (1 mm diameter) in the center of the acrylic board to reduce the friction caused by the rotation of lazy arm. The acrylic cylinder arena was used to eliminate the interference of any air movements on insect flight. Two infrared cameras (Hikvision, Hangzhou, China) were used to record the flight behavior of insects on vertical and horizontal planes. The video data acquisition card was equipped with USB interface to facilitate the setup and dismantling of the tracking platform. The tracking platform was checked before and after each experiment to ensure smooth rotation of the directional pointer and normal image display of the computer. The time interval was set at *t=T/N* (*T* was the tracking time and *N* was the number of position data points acquired within the tracking time).

### Video data acquisition and target position processing

The flight behavior of armyworm moths was monitored on a video screen, and recorded to DVD in AVI format during trials at a resolution of 640×480 with 25 frames per second. The moving target tracking software consisted of tracking interface layer and CamShift tracking algorithm (Bradski, 1998). It was programmed based on Open Source Computer Vision (Open CV) development library and Microsoft Foundation Class (MFC) under the environment of Visual C++ 6.0. The Open Source Computer Vision Library is a cross-platform computer library of a series of C functions and some C++ classes that can be used to implement many common image processing and computer vision algorithms. The black arrow marked on the direction pointer was detected using the Camshift algorithm and the position of the arrow mark was displayed on the tracking interface simultaneously. The CamShift tracking algorithm uses the target's color information to perform continuous tracking and recognition (Shen et al., 2012). The displayed position data of each frame image was saved to an excel spreadsheet after the tracking ends.

### Experiments with the flight simulation system

Flight simulator trials were conducted indoors at Institute of Electrical Engineering, Chinese Academy of Sciences, in Beijing (latitude 40.0° N, longitude 116.3° E). The circadian clock of insects had been altered before the flight trial by reversing the day and the night photoperiod in an incubator environment chamber for two weeks (5th instar to adults). Before the flight trials, the individual moths were mildly anesthetized using ether. The scale-hairs on the dorsum were removed, and the dorsum was glued onto the bent tip of the installation rod of the flight simulator perpendicular to the body axis (Fig. 6). The flight behavior was assayed during the artificial nighttime in the flight simulator in complete darkness. When a moth began to vibrate its wings at the beginning of the night phase, it was connected to the flight simulator via the coupler (Fig. 6) with the head initially pointed to the geomagnetic north pole. Each moth was held in the non-metallic holding cage positioned within the coil system for 1 h to acclimate them to the trial conditions. Each moth was videoed using the horizontal camera and vertical infrared cameras for 30 min in the darkness and the first active 10 min of flight was used for data processing (Fig. 6). The head direction was recorded from each video frame and the distribution of heading directions for each individual moth was analyzed by using the records of whole video frames. It is noteworthy that the WMF and SMF experiments were carried out in different time points, the two GMF moth groups would be different in terms of mean direction angles. A total sample of 195 insects was used for the flight experiments with 63 successful and 132 failed test moths.

### Data analysis

The initial position of the moth was set as the axis center. The directional angles of flight were calculated according to the *x*-*y* data. The video tracking information could be obtained in real time and vectors were used to save the video position data due to the large number of tracking frames. The flight trajectories were calculated in MATLAB (Mathworks) using coordinate transformation and mathematical modeling of tracking position data for subsequent statistical analysis.

Circular statistical analyses (descriptive and comparative) were performed using a circular statistics toolbox for MATLAB 2013b (Berens, 2009). The indexes, including mean resultant vector direction, median resultant vector direction, resultant vector length (*r*), and variance were calculated. The closer *r* is to one, the more concentrated the data sample is around the mean direction. The median direction is indicative of central tendency. The variance is indicative of the spread in a data set. Rayleigh's test was used for analyses of mean common orientation, and Watson–Williams test was used for comparisons of different magnetic field groups.
